# Mass Spectrometry of Polymer Electrolyte Membrane Fuel Cells

**DOI:** 10.1155/2016/6097285

**Published:** 2016-11-29

**Authors:** Viktor Johánek, Anna Ostroverkh, Roman Fiala, Andrii Rednyk, Vladimír Matolín

**Affiliations:** Department of Surface and Plasma Science, Charles University in Prague, V Holesovickach 2, 180 00 Prague 8, Czech Republic

## Abstract

The chemical analysis of processes inside fuel cells under operating conditions in either direct or inverted (electrolysis) mode and their correlation with potentiostatic measurements is a crucial part of understanding fuel cell electrochemistry. We present a relatively simple yet powerful experimental setup for online monitoring of the fuel cell exhaust (of either cathode or anode side) downstream by mass spectrometry. The influence of a variety of parameters (composition of the catalyst, fuel type or its concentration, cell temperature, level of humidification, mass flow rate, power load, cell potential, etc.) on the fuel cell operation can be easily investigated separately or in a combined fashion. We demonstrate the application of this technique on a few examples of low-temperature (70°C herein) polymer electrolyte membrane fuel cells (both alcohol- and hydrogen-fed) subjected to a wide range of conditions.

## 1. Introduction

Direct alcohol fuel cells (DAFCs) [1, 2] attracted a lot of attention in the last few decades for their great potential for direct production of electricity from renewable fuels without the use of an additional reforming reactor. Despite the fact that the concept of direct alcohol fuel cells proved viable, there remain many issues to be solved before these environmentally friendly power sources may be widely accepted as being sufficiently efficient, reliable, and affordable. One of the approaches in DAFC development is chemical analysis of its products and their connection with other operational parameters. It can provide very valuable information in understanding origins of cell efficiency losses, evolution of environmentally harmful byproducts, poisoning of FC catalysts or other potential sources of FC damage (corrosion and etching of gas diffusion layers or flow fields, degradation of membrane, degradation of seals, etc.). For instance, in direct methanol fuel cell (DMFC) often side production of carbon monoxide, formaldehyde, formic acid, or hydrogen peroxide is a serious issue limiting the lifetime of the membrane electrode assembly (MEA) [3, 4]. Another example is the well-known phenomenon of fuel cross-over [3] which is the main cause of relatively low energy density attainable with current DAFCs as compared to their hydrogen-fed competitors (H_2_ PEMFCs). And last but not least, the release of carbon monoxide or dioxide in hydrogen PEMFC and generation of HF or CF_*x*_ species in FCs with teflon based (e.g., Nafion) membranes are clear indicators of MEA degradation. This issue becomes even more serious under higher cell potentials, which is a typical situation of FC running in an inverted regime for electrolysis.

In electrocatalysis so-called differential electrochemical mass spectrometry (DEMS) [5, 6] is sometimes used to detect volatile products of electrochemical reactions in real time. Typical setup consists of a membrane, a frit, or a pinhole separating the electrochemical cell from a vacuum vessel housing a mass spectrometer. More sophisticated instruments use a differentially pumped vacuum system with one or more pumping stages [6]. Such systems are particularly important in online analysis of fast (subsecond) processes [7] such as those occurring under transient regimes or during chemical oscillations. Main advantages of the DEMS sensors are fast response time and high sensitivity.

DEMS has also been adopted specifically for the detection of products of cells by using a membrane interface [6] or integrating the mass detector into the flow field [8]. It provides a great tool for monitoring fast transient processes occurring in an operating fuel cell. On the other hand, in most cases the separation of the electrolyte phase from the vacuum is maintained by a porous membrane which needs to be, at the same time, permeable enough to allow the reaction products to enter the mass spectrometer detection chamber. This also implies that the DEMS method is limited to substances which are volatile enough to efficiently vaporize into the vacuum system.

An alternative approach in FC research is an in-line setup in which the distribution of products is monitored by sampling the contents of the fuel cell exhaust line through a membrane (typically for liquids), capillary (typically for gaseous products), or other flow-limiting device. Chromatographic methods such as gas chromatography or high-performance liquid chromatography are commonly utilized in fuel cell exhaust online analysis. Compared to these techniques mass spectrometry is, in general, significantly faster method which allows continuous monitoring of the composition of FC product stream. Unlike standard DEMS the in-line method is not limited to volatile compounds as the transfer of reactants to and products from the reaction cell is ensured by the forced flow which can be easily controlled externally, unless for the species which would bind strongly to the investigated catalytic material or to the surface of the respective gas diffusion layer supporting the catalyst. Even with the simple and relatively robust design presented in this work valuable results can be obtained for slower processes or under steady-state conditions. The versatility of this setup equipped with adjustable dosing valve and a cold trap is that it can cover a very wide range of concentrations and pressure conditions and allows quick and easy switching between FC assembly (for electrochemistry studies) and catalytic flow reactor (for pure catalytic studies).

In this report recent examples of the application of mass spectrometry in real-time analysis of vapor-fed direct alcohol fuel cell [9, 10] and hydrogen PEMFC [2] operated in reverted (electrolytic) regime [11] are presented. The same approach can be used to analyze standard liquid-fed FCs as long as it is provided that the liquid (or potentially condensing) substances are efficiently removed from the exhaust stream, for example, by a cold trap as in our experimental setup.

## 2. Materials and Methods

**Figure 1:**
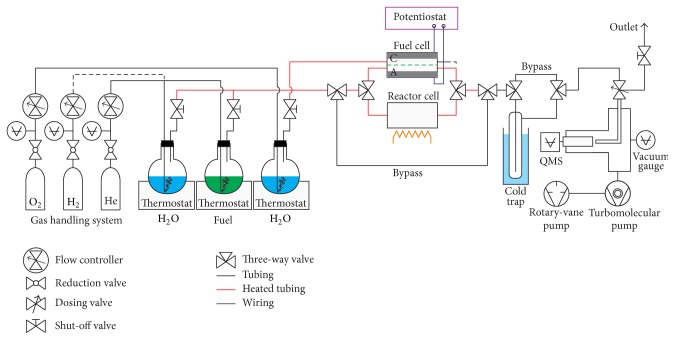
Schematic depiction of the experimental setup used for mass spectrometry of fuel cells.

An experimental fuel cell was connected to a laboratory microreactor system schematically depicted in Figure 1. Its design allows on-the-fly switching between fuel cell, flow reactor, and bypass by directing the gas (or vapor) feed through the respective line.

Methanol and water vapor sources were realized by bubbling helium buffer gas (acquired from Linde Gas, 4.6 purity) through independent saturators filled with liquid fuel (methanol or ethanol, Penta, 99.5% purity) and deionized ultrapure water (resistivity ≥ 18.2 MΩ·cm at 25°C), respectively. The saturators were immersed in a thermostat to maintain constant partial vapor pressures with independently controlled temperatures for water (343 K) and methanol (315 K). In the case of hydrogen-feed the fuel was mixed with water vapor by bubbling hydrogen (Linde Gas, 5.0, ≤0.5 ppm hydrocarbon impurities) without any additional buffer gas. In both types of experiment, pure oxygen (Linde Gas, 5.0) was supplied to the cathode side after humidification in a third saturator filled with deionized water at 343 K at 30 sccm flow rate. All the measurements presented herein were carried out with the fuel cell assembly thermally stabilized at 343 K. The flow rates of He, O_2_, and H_2_ were precisely adjusted by mass flow controllers (Alicat Scientific). All stainless-steel tubing between the saturator and the reaction cell was heated to about 360 K to prevent condensation of methanol or water vapor before reaching the cell. A flow reactor with precise temperature controller installed in parallel with the fuel cell was available for reference reactivity studies of our catalysts under potential-free conditions.

The downstream gas mixture (from either anode or cathode exhaust line) was sampled through a precise manual dosing valve with integrated shut-off valve (Leybold EV 016 DOS AB), directed towards the top of spectrometer head by a stainless-steel 1/8′′ ID tube, and monitored online by a quadrupole mass spectrometer (QMS, Pfeiffer Prisma 200). The QMS was enclosed in a small stainless-steel bakeable vacuum chamber with <10^−6^ Pa base pressure, evacuated by a 70 l/s turbomolecular pump in series with a rotary-vane pump. A cold trap (laboratory glass condenser immersed in a Dewar flask filled with ethanol cooled typically to 190–200 K or 260 K in DAFC or PEMFC experiments, resp.) was put into the line to prevent the excess water or unreacted methanol vapor from entering the vacuum chamber with QMS or blocking the dosing valve. The relative molecular concentrations of the main products H_2_ (*m*/*z* = 2 amu), CO (28 amu), CO_2_ (44 amu), and O_2_ (32 amu) were calculated from QMS current signals using sensitivity factors obtained experimentally by calibration with pure gases. Other masses have been monitored as well to detect all potential byproducts and to allow assignment of parent molecules to some ambiguous mass signals, typically 4, 12, 14, 15, 16, 17, 18, 22, 29, 30, 31, 34, 40, 45, 46, 60, and 75 amu. The QMS signals have been postsynchronized with electrical measurements measured by potentiostat, taking into account the delay due to the transport of gases from the fuel cell to the QMS analytical chamber.

Regarding the QMS calibration procedure, the following factors have been taken into account:

- Relative sensitivity of the reference vacuum pressure gauge for a given gas molecule: if the absolute pressure gauge (such as diaphragm pressure transducer) is used, no further correction is required
- Correction of the partial pressure of a given gas to account for the pressure gradient inside the vacuum chamber to obtain the partial pressure at the QMS head: the actual procedure depends on the particular geometry, with the main factor typically being the pumping speed (which, again, generally differs for each gas)
- Intrinsic sensitivity of the QMS, calculated from the measured relation between the QMS current for a given mass and the partial pressure of the respective species at QMS head: in the cases where the sensitivity was not (or could not be) determined experimentally, the sensitivity value can be estimated based on another (ideally chemically similar compound) which we know the sensitivity of, using the ratio of their electron ionization probabilities
- Correction to different relative dynamic viscosity in the flow channel of the dosing valve for each species analyzed: typically these values are provided by the manufacturer of the respective valve or they can be found in literature [12, 13]
- Molar weight of a given molecule in order to convert the pressure-related values to molar concentrations

In our case, the calibration was done separately for different dosing valve settings, spanning almost 3 orders of magnitude (air equivalent flow rates 2 × 10^−5^–1 × 10^−2^ mbar·l·s^−1^). By making use of the proper valve setting for each particular situation the dynamic range of the instrument can be greatly extended.

Membrane electrode assembly (MEA) of the fuel cell with an active area of 1 cm^2^ was formed from a 50 *μ*m thick proton conductive Nafion membrane (DuPont Inc., Nafion NR-212, perfluorosulfonic acid-PTFE copolymer) sandwiched between the catalyzed anode and cathode by pressing at room temperature. For DAFC experiments commercial electrodes (Alfa Aesar) consisting of nano-GDL (gas diffusion layer) supports (260–280 *μ*m thick) coated with Pt nanodispersed catalyst (2 mg/cm^2^ nominal loading) were used on both cathode and anode side. Two carbon gas distributor plates (FC-01-02 from ElectroChem with 1 cm^2^ active area) without any pretreatment were used as a fuel cell body and clamped together with 3 Nm torque per screw (resulting in 15 bar pressure on MEA).

For the electrolysis experiments a carbon free cathode setup with Pt nanocatalyst supported on carbon-based substrates was used. Amorphous carbon (further denoted as a-C) and carbon nitride (CN_*x*_) thin films were prepared by dc magnetron sputtering from a graphite target (Goodfellow, 99.997% purity) directly onto Nafion membrane (Nafion NR-212) using a commercial Modular High Vacuum Coating System MED020 (BALTEC). The depositions were carried out in argon or nitrogen atmosphere to obtain a-C or CN_*x*_, respectively, with partial pressure of the process gas of approx. 0.8 Pa. These layers with thickness of 200 ± 20 nm [14] were covered with Pt nanoparticles (10 nm average thickness) by dc magnetron sputtering from Pt target (Goodfellow, 99.95% purity) in pure argon, using a magnetron sputtering system of our proprietary construction. The measurements were performed using titanium flow field plates coated with TiN protective layer and a titanium mesh from Dexmet was used as gas diffusion layer.

## 3. Results and Discussion

### 3.1. Anode Exhaust Analysis of Direct Ethanol Fuel Cell

In the first section we demonstrate the electrochemical analysis of an alcohol vapor feed fuel cell operating in a wide range of conditions.

**Figure 2:**
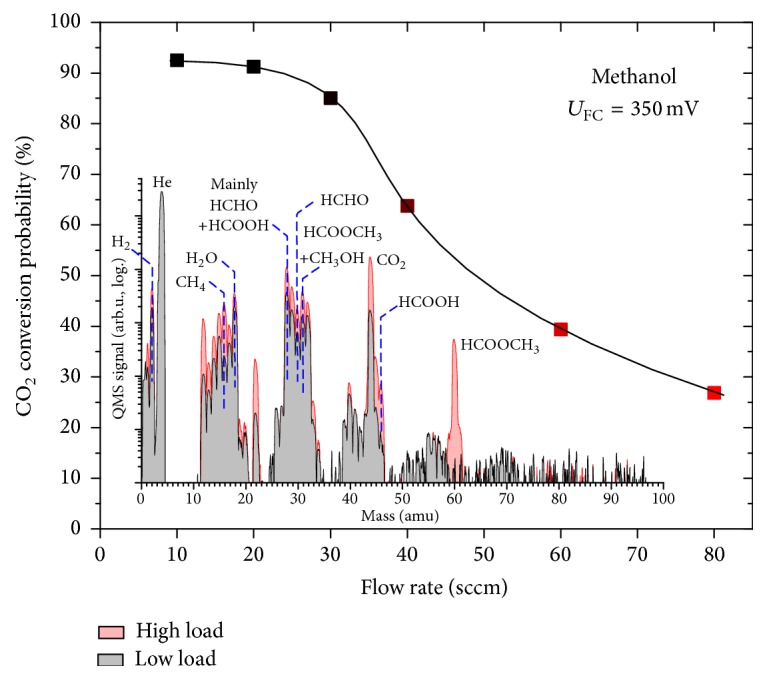
Dependence of the probability of methanol-to-CO_2_ conversion on the flow rate of fuel delivered to the fuel cell anode. Methanol concentration was 2 M, cell potential 350 mV. Inset: comparison of typical mass spectra of DMFC anode exhaust acquired at low and high fuel load for the same methanol-fueled cell.

The main curve in Figure 2 presents the dependence of the probability of methanol conversion to CO_2_ on the total flow rate of the He + H_2_O + CH_3_OH fuel mixture. The ratio of methanol to water in the feed was set to represent the molar equivalent of 2 M liquid solution; the external potential of the cell was kept at constant 350 mV.

The relation between the relative molecular concentration of methanol in the methanol + water mixture *c*_MeOH_ and liquid equivalent of fuel molarity *η*_MeOH_ (moles per volume) of methanol diluted in water can be expressed as

(1a) ηMeOH=1MMeOH/ρMeOH+MH2O/ρH2O·1/cMeOH−1

or, expressed inversely,

(1b) cMeOH=1ρH2O/MH2O·1/ηMeOH−MMeOH/ρMeOH+1,

where *ρ*_MeOH_, *M*_MeOH_, and *ρ*_H_2_O_, *M*_H_2_O_ are mass concentrations and molar masses of methanol and water, respectively. For *η*_MeOH_ = 2 M we get relative methanol concentration in the vapor stream *c*_MeOH_ = 0.0378.

The methanol volume flow rate is determined by the flow rate of the carrier gas (He) to methanol saturator (*f* ^He^_MeOH_) and by methanol relative concentration in the gas phase mixture which depends on its partial vapor pressure and is thus temperature dependent:

(2) $$\begin{array}{c} f_{MeOH}\left(\;T_{MeOH}\;\right)={f^{He}}_{MeOH}\cdot \frac{P_{MeOH}\left(T_{MeOH}\right)}{P_{tot}}, \end{array}$$

where *P*_MeOH_(*T*_MeOH_) is partial pressure of methanol vapor at given temperature *T*_MeOH_ and *P*_tot_ total pressure in the feed gas line (*P*_He_ + *P*_H_2_O_ + *P*_MeOH_). In order to produce the desired fuel composition the stream of carrier gas has to be split between the two saturators. By combining with analogous equation for water saturator we can express the required He flow rate *f* ^He^_MeOH_ by methanol relative molecular concentration in the methanol + water mixture (*c*_MeOH_) and the total flow rate (*f*_tot_) to anode as

(3) fHeMeOH=ftot1−cMeOH·PMeOHTMeOH/cMeOH·PH2OTH2O+1

which can be for small methanol concentrations (*c*_MeOH_ ≪ 1) and *P*_MeOH_ ≳ *P*_H_2_O_ simplified to

(4) $$\begin{array}{c} {f^{He}}_{MeOH}\approx f_{tot}\cdot c_{MeOH}\cdot \frac{P_{H_{2}O}\left(T_{H_{2}O}\right)}{P_{MeOH}\left(T_{MeOH}\right)}. \end{array}$$

By substituting (3) to (2) we obtain

(5) fMeOHTMeOH,TH2O=ftotPtot·1PMeOHTMeOH+1−cMeOHcMeOH·PH2OTH2O−1

or, if expressed by relative vapor pressures *σ*_*x*_ = *P*_*x*_/*P*_tot_,

(6) fMeOHTMeOH,TH2O=ftot1/σMeOHTMeOH+1−cMeOH/cMeOH·1/σH2OTH2O.

To calculate vapor pressures of both substances we can use the empirical formulas based on Clausius-Clapeyron equation [15]; thus for *P*_He_ = 101.5 kPa

(7) σMeOHTMeOH=1.3135×10−3·10−2001.663/TMeOHK+8.8017,σH2OTH2O=1.3135×10−3·10−2228.799/TH2OK+8.8535,

where *T*_MeOH[K]_ and *T*_H_2_O[K]_ are temperatures of methanol and water, respectively, expressed in Kelvins.

For *f*_tot_ = 80 sccm, *T*_MeOH_ = 315 K, *T*_H_2_O_ = 343 K, and *η*_MeOH_ = 2 M the resulting requirement for *f* ^He^_MeOH_ is 2.35 sccm.

The inset of Figure 2 shows the example of two mass spectra of the DMFC anode exhaust acquired in the 0–100 amu range for the unit operating at a low fuel load (10 sccm, average output power density of ~10 mW·cm^−2^) and a relatively high fuel load (80 sccm, average output power density of ~40 mW·cm^−2^). Most common species detected during the operation of the DMFC at the anode are carbon dioxide (CO_2_, principal mass 44 amu), formaldehyde (HCHO, 30 amu), formic acid (HCOOH, 46 amu), methyl formate (HCOOCH_3_, 60 amu), water (H_2_O, 18 amu), hydrogen (H_2_, 2 amu), and methane (CH_4_, 16 amu). In some cases (e.g., high cell potentials) dimethoxymethane (H_2_C(OCH_3_)_2_, 75 amu) was also observed. The peak intensity for any given mass is comparable between different experiments but different masses cannot be compared mutually since these are raw QMS spectra. For quantitative evaluation the above described concentration calculation has to be made. Furthermore, it should be taken into account that the signal of some substances is reduced due to their condensation in the cold trap (e.g., HCOOH, HCHO, or HCOOCH_3_). Only sufficiently characteristic masses were labeled in Figure 2; the peaks at 12, 15, and 29 amu as well as some others are too ambiguous for useful analysis.

Now we identify the origin of the observed species. It is well known [1, 2, 16] that at moderate conditions the majority of methanol in water-rich environment is oxidized to CO_2_ via the ideal anodic half-reaction:

(R1) $$\begin{array}{c} {CH}_{3}OH+H_{2}O⟶{CO}_{2}+6H^{+}+6e^{-} \end{array}$$

Under more intensive exposure of the FC catalyst to the fuel stream a gradually increasing preference towards formation of formic acid can be observed as a result of incomplete methanol electrooxidation:

(R2) $$\begin{array}{c} {CH}_{3}OH+H_{2}O⟶HCOOH+4H^{+}+4e^{-} \end{array}$$

which is, in the excess of methanol, typically followed by the formation of methyl formate via

(R3) $$\begin{array}{c} HCOOH+{CH}_{3}OH⟶{HCOOCH}_{3}+H_{2}O \end{array}$$

As the fuel mixture becomes even more methanol-rich, the lack of water can eventually lead to the following reaction route:

(R4) $$\begin{array}{c} {CH}_{3}OH⟶HCHO+2H^{+}+2e^{-} \end{array}$$

generating formaldehyde and only 2 electrons per methanol molecule, further lowering the output performance of the fuel cell. The formaldehyde tends to bind to another available methanol molecule via

(R5) $$\begin{array}{c} HCHO+2{CH}_{3}OH⟶H_{2}C{\left(\;{OCH}_{3}\;\right)}_{2}+H_{2}O \end{array}$$

yielding dimethoxymethane species.

So there exists an optimal working regime of the fuel cell in terms of fuel composition above which a decay of its efficiency takes place. In our case (Figure 2) the loss of performance is the result of growing feed flow rate while the methanol content remains constant at relatively low value (2 M). This suggests that the dissociation of water is probably the rate limiting step of the methanol conversion for this type of catalyst [16].

**Figure 3:**
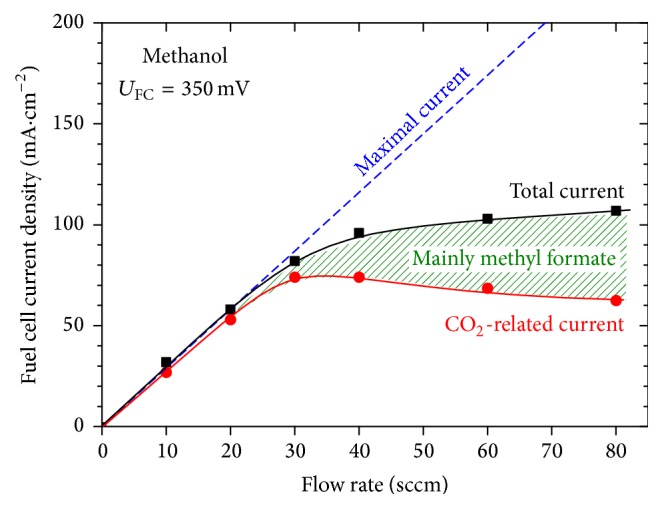
DMFC current as a function of total fuel mixture flow rate. Methanol concentration in the feed was 2 M, cell potential 350 mV. The dashed blue line represents the ideal line of maximal current.

In Figure 3 the dependence of current density generated by the DMFC in the same experiment as above is plotted for the feed flow rate of *f*_tot_ = 10–80 sccm. The total current was measured at the output by a potentiostat while the CO_2_-related portion of the total current was calculated from the amount of released CO_2_ (presented in Figure 2) assuming all 6 electrons per molecule are collected in the circuit.

Under assumption that there is no other limiting factor such as lack of water or lack of oxygen at cathode we can calculate the theoretical value of maximum current attainable by the methanol-fed cell for a given set of conditions. Faraday law defines the relation between reactant consumption (quantified by fuel molar flow *q*_MeOH_ = d*N*_MeOH_/d*t*) and electron current (*I*_max_) as

(8) $$\begin{array}{c} I_{max}=6\cdot F\cdot q_{MeOH} \end{array}$$

because 6 electrons are generated from each methanol molecule; *F* is Faraday constant. The volume flow rate can be converted to the molecular flow rate via molar gas volume at STP (*V*_*m*_ = 22.414 l·mol^−1^) as

(9) $$\begin{array}{c} q_{MeOH}=\frac{f_{MeOH}}{V_{m}} \end{array}$$

(the use of the STP value is appropriate here because the carrier gas was dosed by a flow controller working at 1 atm pressure and room temperature). Hence, by evaluating all constants as a single value and combining the above equations with (6) we obtain

(10) ImaxA≈0.43047·ftotsccm1/σMeOHTMeOH+1−cMeOH/cMeOH·1/σH2OTH2O.

Thus for the above values of flow, molarity, and temperatures we get the maximum current *I*_max_ = 232 mA at 80 sccm; see the corresponding blue dashed line in Figure 3.

As could be expected, the more intensive the fuel supply is, the more the total power can be generated by the fuel cell, following the ideal line of maximal current on the left side of the plot. However, the efficiency of the device drops substantially above certain threshold (in this case, around 30 sccm) indicating that the cell runs outside its optimal working regime, in accord with the above discussion. The analysis of the mass spectra (see also the inset of Figure 2) reveals that the difference between the two values of current density (total and CO_2_-related) originates in major part from the incomplete electrooxidation of methanol to methyl formate, yielding only 4 electrons per molecule (reaction R3) and, moreover, consuming an additional methanol molecule which is thus effectively removed from the feed.

**Figure 4:**
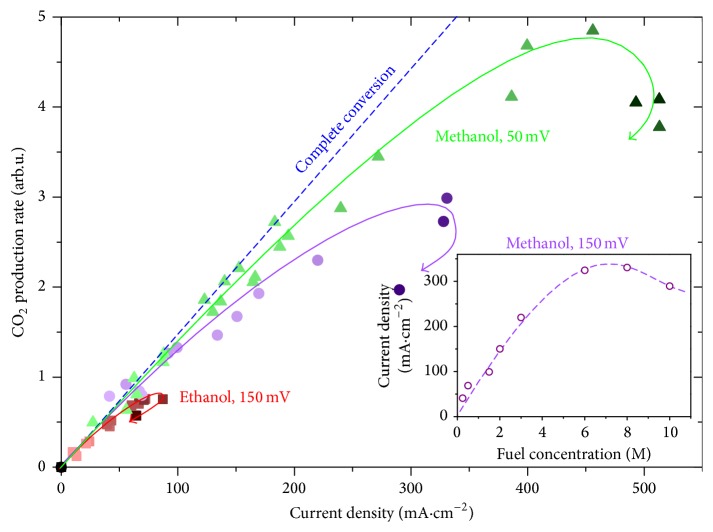
Correlation between current density generated by fuel cell and CO_2_ production at its anode for various fuel concentrations between 0.25 and 10 M (methanol) and 0.25 and 14 M (ethanol); total flow rate was 25 sccm. The color intensity of experimental points reflects the fuel density. Blue dashed line represents complete conversion of alcohol molecule to CO_2_. Inset: current density of the methanol-fed FC at 150 mV as a function of fuel molarity.

The similar behavior is observed if the fuel supply is varied through its concentration in otherwise constant upstream flow rate. In Figure 4, our test FC unit was examined by feeding the anode channel with the mixture of gaseous water and methanol with variable mutual ratio, whereas the total flow rate of the mixture was held constant at 25 sccm. The calculated ideal line of complete methanol-to-CO_2_ conversion has been marked in the graph for reference. Ethanol fueled FC operated at the same conditions is also shown in Figure 4 for comparison.

The variation of methanol molar content from 0.25 to 10 M leads to output current density span of approx. 15–330 mA·cm^−2^ at 150 mV cell potential (and 15–500 mA·cm^−2^ at 50 mV). The molarity of the fuel mixture in Figure 4 is represented by the color intensity of the plotted experimental points. As can be expected, at low or moderate concentrations the cell power output is proportional (or nearly proportional) to the amount of delivered methanol. Further increasing the fuel density is, however, associated with gradual decrease of methanol conversion efficiency which even leads to a saturation in current density at some point; that is, making the fuel mixture more rich does not gain any more current (see also inset of Figure 4). In fact, the net performance of the unit may become even worse in the terms of both reaction efficiency and total current gain, which is indicated by the descending sections of the curves in Figure 4. We assume this is mainly due to instability issues which can occur, for example, as a result of temporary blocking of catalyst surface by reaction intermediates or by membrane drying in the water-lean environment. In the long term, running far above optimal conditions or at higher loads may even lead to substantial decrease of the device lifetime [1, 17].

From the comparison of 50 mV and 150 mV methanol curves one can also see an illustration of the general trend that at lower cell potentials the tolerance for more fuel rich feed is higher. This is commonly attributed to voltage dependence of the fuel cross-over through the proton exchange membrane (via electroosmotic drag [18, 19]), which leads to the parasitic electrooxidation of methanol at the cathode and can significantly reduce the overall cell efficiency [3].

A qualitatively similar behavior is observed with diluted ethanol fuel. In this case, however, varying the molar concentration from 0.25 to 14 M resulted in current densities not exceeding 100 mA·cm^−2^ at 150 mA; hence the saturation occurs at considerably lower currents compared to methanol-fueled FC at the same potential. This can be partly explained by the fact that 3 water molecules are required for the full conversion of each ethanol molecule according to the anodic half-reaction

(R6) $$\begin{array}{c} C_{2}H_{5}OH+3H_{2}O⟶2{CO}_{2}+12H^{+}+12e^{-} \end{array}$$

while releasing only twice as many electrons, so that 50% more water per current unit is needed. This is again in accord with the idea of water dissociation being the rate limiting step of the catalytic electrooxidation of alcohol at anode. There has to be, however, additional restrictive factors in play here (e.g., catalyst poisoning by intermediate products of ethanol oxidation [20] or higher energetic cost of breaking C–C bond) but their exact elucidation is outside the scope of this article.

### 3.2. Electrochemical Corrosion of Fuel Cell

The operation regime of fuel cells can be switched between the regular mode of electricity generation (normal FC operation) and the electrolysis mode (hydrogen and oxygen production). This concept is the basis of so-called reversible, or regenerative, fuel cells (RFC) which are devices optimized to be efficient enough in both directions. In hydrogen-fueled PEMFC the electrolytic process splits water molecule to oxygen molecule and two protons which are transported through the membrane and recombine to hydrogen molecule at the cathode (note that FC-mode anode becomes cathode in the electrolysis regime) while consuming two electrons which need to be supplied from an external source. However, the presence of oxidative environment and relatively high external voltage can lead to other side reactions, potentially involving components of the device. In particular, carbon, commonly used as a material for electrodes, GDLs, and catalyst supports, turns out to be prone to electrooxidation. In some cases, we detected loss of FC performance due to depletion of the catalytic material via oxidative removal of carbon even under normal power generation operation at higher current densities.

We analyzed the resistance of 200 nm thick magnetron sputtered amorphous carbon (a-C) and carbon nitride (CN_*x*_) layers covered with Pt nanoparticles (10 nm average thickness) against oxidative etching in electrolysis mode. The catalysts were deposited directly onto a Nafion membrane and incorporated into both sides of MEA.

**Figure 5:**
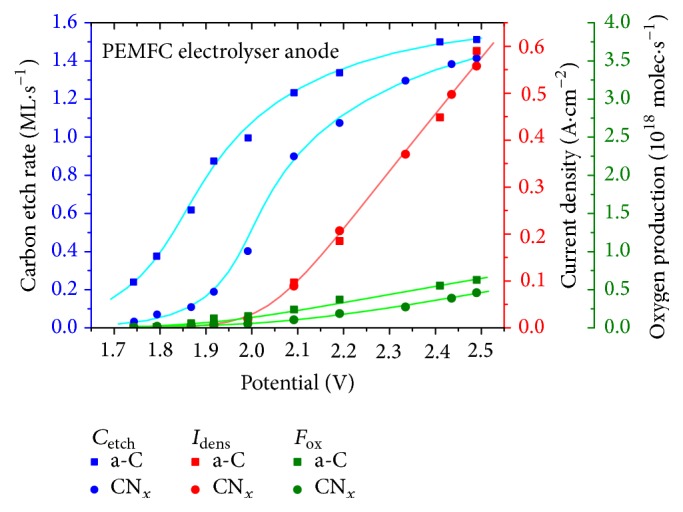
Dependence of carbon etch rate at the FC cathode (*C*_etch_, blue), total current density (*I*_dens_, red), and oxygen production rate (*F*_O_, green) of PEMFC on its potential for Pt catalyst supported on amorphous carbon (squares) and carbon nitride (circles).

The results obtained at the PEMFC electrolyser anode for both types of catalytic layers are presented in Figure 5. The PEMFC was operated at 343 K and cathode and anode fed with humidified (H_2_O at 343 K) hydrogen (40 sccm) and oxygen (30 sccm) as in normal FC operation. The inverted cell voltage was increased stepwise between 1.75 and 2.5 V and the resulting electric current and mass signals of selected gases were recorded simultaneously. Eventually, only the 44 amu signal (CO_2_) was used in carbon balance analysis since no discernible change in other carbon-containing gaseous products (such as CO or hydrocarbons) has been detected.

With the calibration described in Materials and Methods the molecular flow rate of CO_2_ can be determined from the 44 amu QMS signal and converted to the amount of carbon atoms removed per unit time. Using a hexagonally arranged carbon layer as a representative structure of carbon the atomic density of the etch rate can be expressed in terms of carbon monolayers per second (ML·s^−1^) as in Figure 5.

Just to give an idea to what extent this process affects the lifetime of the fuel cell, our 200 nm thick catalytic Pt/a-C layer would be completely reacted off after approx. 10 minutes at 2 V cell potential (assuming volume density close to graphitic structure).

As can be seen in Figure 5, above the onset observed at the potential of about 1.9 V the current density is directly proportional to the external cell voltage and follows exactly the same curve regardless of the catalyst support material. The amount of oxygen released as a result of the electrolytic process at the anode (green points in Figure 5, calculated from 32 amu mass signal) is also proportional in this linear region. The quantification of the O_2_ signal versus supplied current yields total water electrolysis efficiency varying between approx. 17 and 32% (for a-C) and 13 and 20% (for CN_*x*_), respectively. The efficiency is quite low, presumably because the unit was not operated in ideal conditions for electrolysis but rather under normal FC conditions with reverted voltage, affecting the local chemical potentials on both electrodes.

On the contrary to the current dependence, a-C and CN_x_ exhibit different behavior in regard to susceptibility to carbon etching; nitrided carbon is stable up to about 1.7 V and provides up to fivefold increase in resistivity against electrooxidation (at potentials ≲ 1.9 V) compared to pure carbon. The voltage dependence of carbon etch rate of both materials is nonlinear and does not scale with current density, especially at higher potentials where it seems to be approaching a saturation value. We suppose this might be due to mass transfer (diffusion) limitation phenomena in the removal of generated CO_2_ from the porous material of the catalyst.

## 4. Conclusions

Polymer electrolyte membrane fuel cells based on carbon-supported platinum nanocatalysts were subjected to real-condition tests in both normal and inverted regime. A relatively simple mass spectrometry system has been used for online monitoring of composition of FC products and correlated with electrical parameters measured by a potentiostat. The experimental setup allows independent control over several working parameters, namely, type of fuel (from gases to vapors), fuel-to-oxygen stoichiometry, humidity, cell temperature, flow rates, pressure in the feed, power draw, and cell potential. The formulas for quantitative evaluation of the obtained experimental data have also been provided.

We have demonstrated the use of this technique on two examples:

(i) Response of alcohol (methanol and ethanol) fueled FCs to various intensities of fuel supply and output power: it is shown that when the Pt-Pt FC is subjected to more harsh conditions (higher fuel concentration, fuel flow rate, or current draw) the alcohol oxidation at anode deviates from ideal reaction pathway, producing side products (primarily methyl formate in the case of methanol) and losing its fuel-to-electricity conversion efficiency
(ii) The fact that hydrogen fueled PEMFC with Pt/a-C or Pt/CN_x_ electrodes operated in reverted mode is susceptible to electrochemical corrosion, leading to depletion of catalytic material: at FC cathode the carbon support is reacted away primarily to CO_2_ while Pt nanoparticles are probably washed away by water present in the feed. Nevertheless, the CN_*x*_-based layer provides somewhat higher resistance to oxidation as compared to a-C. The carbon etch rate increases with cell potential

This report provides a methodological overview of the practical electrochemical analysis of operating fuel cells rather than a systematic fundamental study of the behavior of a particular FC system. More thorough presentation and discussion of the investigation of fuel cells with different types of catalysts will be the subject of following publications.
